# Implementation of mandatory opioid prescribing limits in North Carolina: healthcare administrator and prescriber perspectives

**DOI:** 10.1186/s12913-021-07230-5

**Published:** 2021-11-03

**Authors:** Natalie A. Blackburn, Elizabeth Joniak-Grant, Maryalice Nocera, Samantha Wooten Dorris, Nabarun Dasgupta, Paul R. Chelminski, Timothy S. Carey, Li-Tzy Wu, David A. Edwards, Stephen W. Marshall, Shabbar I. Ranapurwala

**Affiliations:** 1 University of North Carolina Injury Prevention Research Center, Chapel Hill, NC 27516 USA; 2 Department of Health Behavior, Gillings School of Global Public Health, University of North Carolina at Chapel Hill, Chapel Hill, NC USA; 3 Office of Research, Innovations, and Global Solutions, Gillings School of Global Public Health, University of North Carolina at Chapel Hill, Chapel Hill, NC USA; 4 Department of Medicine, School of Medicine, University of North Carolina at Chapel Hill, Chapel Hill, NC USA; 5 Cecil G. Sheps Health Center for Services Research, University of North Carolina at Chapel Hill, Chapel Hill, NC USA; 6 Department of Psychiatry and Behavioral Sciences, Duke University Medical Center, Durham, NC USA; 7 Department of Medicine, Division of General Internal Medicine, Duke University Medical Center, Durham, NC USA; 8 Department of Anesthesiology, Vanderbilt University Medical Center, Nashville, TN USA; 9 Department of Epidemiology, Gillings School of Global Public Health, University of North Carolina at Chapel Hill, Chapel Hill, NC USA

## Abstract

**Background:**

Recent increases in state laws to reduce opioid prescribing have demonstrated a need to understand how they are interpreted and implemented in healthcare systems. The purpose of this study was to explore the systems, strategies, and resources that hospital administrators and prescribers used to implement the 2017 North Carolina Strengthen Opioid Prevention (STOP) Act opioid prescribing limits, which limited initial prescriptions to a five (for acute) or seven (for post-surgical) days’ supply.

**Methods:**

We interviewed 14 hospital administrators and 38 prescribers with degrees in medicine, nursing, pharmacy, business administration and public health working across North Carolina. Interview guides, informed by the Consolidated Framework for Implementation Research, explored barriers and facilitators to implementation. Interview topics included communication, resources, and hospital system support. Interviews were recorded and transcribed, then analyzed using flexible coding, integrating inductive and deductive coding, to inform analytic code development and identify themes.

**Results:**

We identified three main themes around implementation of STOP act mandated prescribing limits: organizational communication, prescriber education, and changes in the electronic medical record (EMR) systems. Administrators reflected on implementation in the context of raising awareness and providing reminders to facilitate changes in prescriber behavior, operationalized through email and in-person communications as well as dedicated resources to EMR changes. Prescribers noted administrative communications about prescribing limits often focused on legality, suggesting a directive of the organization’s policy rather than a passive reminder. Prescribers expressed a desire for more spaces to have their questions answered and resources for patient communications. While hospital administrators viewed compliance with the law as a priority, prescribers reflected on concerns for adequately managing their patients’ pain and limited time for clinical care.

**Conclusions:**

Hospital administrators and prescribers approached implementation of the STOP act prescribing limits with different mindsets. While administrators were focused on policy compliance, prescribers were focused on their patients’ needs. Strategies to implement the mandate then had to balance patient needs with policy compliance. As states continue to legislate to prevent opioid overdose deaths, understanding how laws are implemented by healthcare systems and prescribers will improve their effectiveness through tailoring and maximizing available resources.

**Supplementary Information:**

The online version contains supplementary material available at 10.1186/s12913-021-07230-5.

## Introduction

Excessive opioid prescribing has been a key factor driving increasing opioid overdose and death rates across the United States [1, 2]. While excessive opioid prescribing has declined in the last decade, evidence suggests that excessive prescribing for post-surgical opioid prescriptions and the leftover opioids remaining in the households of surgery patients are still prevalent [3]. Similar trends have been seen with opioid prescriptions for acute pain in that dosage remain at or close to where they were 10 years ago [4].

**Fig. 1:**
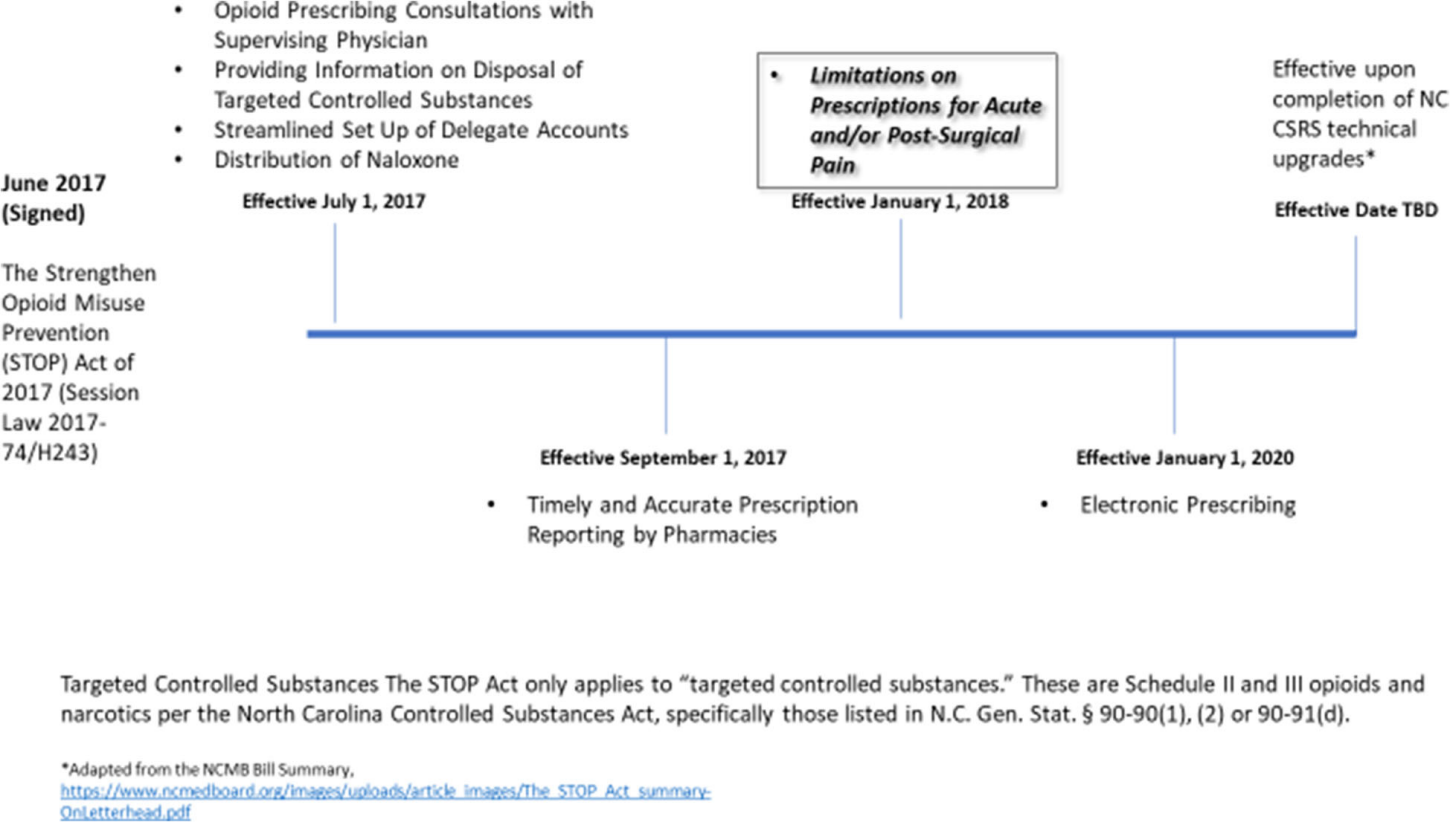
NC STOP Act of 2017 Provisions and Effective Dates

To curb excessive opioid prescribing, the Centers for Disease Control and Prevention (CDC) published opioid prescribing guidelines for chronic non-cancer pain in March 2016 [5]. The CDC guidelines also acknowledged that “long-term pain often begins with the treatment of acute pain,” and that for acute pain, “three days or less will often be sufficient; more than seven days will rarely be needed.” Since then state-level prescribing limits (otherwise referred to as “prescribing caps”) laws to reduce opioid prescribing have proliferated across the country [6]; 31 states have passed laws limiting opioid prescribing in the past 5 years [7]. Whether these types of legislative limits are effective continues to be debated [8], especially as opioid overdose deaths declined in 2018, compared to 2017 but increased in 2019 and 2020 in many states including North Carolina (NC) [9, 10]. Nevertheless, NC passed the Strengthen Opioid Misuse Prevention (STOP) Act in 2017 [11]. The STOP Act includes many provisions to monitor and restrict opioid prescribing as shown in Fig. 1. The focus of our evaluation in this paper is the mandate limiting initial opioid prescriptions for opioid-naïve patients to 5 days for acute pain and 7 days for post-surgical pain. Refills cannot be attached to the initial prescription, but second prescriptions with no limit on duration are allowed. The law does not specify how these limits should be monitored or enforced.

In order to understand the success or failure of policy-based interventions, it is important that the strategies of implementation for these interventions are both identified and critically examined [12]. Prior studies share implementation strategies for evidence-based interventions in healthcare settings [13] in response to internal policy changes and guidelines. However, strategies for implementing laws and mandates that are “required” by a government body have not been addressed. Furthermore, much of the focus of mandates has been on the health outcomes of patients and communities or on compliance [14]; little research has been done to understand how opioid mandates are received by healthcare workers and how such reception, including differences from suggested guidelines, might impact implementation.

Thus, this study aimed to understand the systems, strategies, and resources used by prescribers and healthcare administrators to implement the NC STOP Act prescribing limits. Specifically, we sought to elucidate the practical “hows and whys” of implementation [15] of opioid prescribing limits, a legal mandate, in order to better understand how healthcare legislation can be implemented effectively.

## Methods

### Study design

This qualitative study was conducted in NC from June 2019 to February 2020. We interviewed 14 persons in hospital leadership (hereafter referred to as “Administrators”) and 38 licensed prescribers including physicians, nurse practitioners, and physician assistants (hereafter referred to as “prescribers”). Participants were employed at community hospitals (major teaching, minor teaching, and nonteaching) [16], federal government hospitals, private practices, and regional medical centers located across NC. We used a stratified purposeful sample to recruit prescribers in order to explore variations on experiences from those with different training and patient populations [17, 18]. We employed multiple strategies for sampling prescribers; this included sampling the four most common subspecialties providing outpatient opioid prescriptions for acute and post-surgical pain in NC (i.e. emergency medicine, obstetrics and gynecology, orthopedics, and otolaryngology) [19] using the NC Licensing Board database of physicians and enlisting professional organizations to share details of our study in their newsletters to aid recruitment. We defined eligible prescribers as those licensed in the state of NC who had written at least one opioid prescription for acute and/or post-surgical pain in the past 5 years. Administrators were sampled using the 2018 annual report of the NC Healthcare Association (approximately 118 hospitals); from each hospital we contacted at least one person most suited to speak on implementation of prescribing limits within their hospital system (e.g. Chief Medical Officer, Chief Operating Officer, Chief Nursing Officer, and Chief Quality Officer).

### Study instruments

Two team members (NAB and EJG) developed the two interview guides, one for administrators and one for prescribers. The guides were informed by the Consolidated Framework for Implementation Research (CFIR) which is useful in that it identifies and organizes factors impacting implementation into five domains: outer setting, inner setting, process, intervention characteristics, and characteristics of the individual [20]. We identified the salient constructs within the five domains and adapted relevant questions to fit our topic and policy evaluation context (Table 1).

**Table 1:** Example interview questions for administrators and prescribers based on the CFIR

CFIR Domain	CFIR Construct	Topic area	Questions for Hospital Administrators	Questions for Prescribers
Intervention Characteristics	Complexity	Disruptiveness	How are the prescribing limits affecting your organization’s day-to-day operations?	How have the prescribing limits changed your day-to-day job, if at all?
Outer Setting	Patient Needs & Resources	Patient Communication	Did you communicate implementation of the prescribing limits to patients receiving care at your organization? How so? How have they responded?	Are prescribing limits communicated to patients? How?
Inner Setting	Networks & Communications	Internal Communications	Were the prescribing limits communicated to the prescribers in your organization? Can you tell me more about that process?	In what ways has your organization communicated to you about prescribing limits, whether from a policy developed in-house or based on the NC legislation?
Process	Planning	Hospital Policy/Protocol	Did your organization have a protocol for implementing the prescribing limits? Can you please describe it?	Does your practice/hospital currently have a policy regarding prescribing limits for opioids? Can you describe it to me?
Process	Formally Appointed InternalImplementation Leaders	Key Leaders	Who were the key persons or groups responsible for implementing the prescribing limits in your organization?	In your view, were there key people and/or departments in your organization responsible for implementing prescribing limits?

Additional questions were developed to fill important gaps. Given the number of parts to the NC STOP Act (Fig. 1), questions were written to emphasize the focus on prescribing limits and each interview guide slightly differed based on what role the participants played in the implementation process.

### Ethical approval and reporting

The protocol for this study and all study materials were reviewed and approved by the Institutional Review Board (IRB) at the University of North Carolina, Chapel Hill (study# 18–2437). The study was deemed minimal risk. Informed consent was obtained from each participant after having received oral and written information about the study. Healthcare organizations are not identified by name due to the risk of identifying individual participants. We used the standards for reporting qualitative research (SRQR) when writing our qualitative findings [21] (Additional file 1).

### Data collection

One female interviewer with non-clinical graduate training in public health (NAB) conducted all interviews either at the participant’s office or by phone. Participants provided informed consent to participate and received a $50 gift card for their participation. Interviews lasted approximately 45 to 75 min and were recorded and transcribed. NAB conducted interviews one-on-one with the exception of one administrator interview that included three people from the same hospital, at their request. After the initial interviews in each group NAB met with a second team member (EJG) to review data quality and identify areas where clarification or more information was needed.

### Data analysis

We developed the codebook using a flexible coding approach, which integrates deductive and inductive analytic methods [22]. EJG developed a set of index codes deductively based on the interview guides, CFIR framework, and study objectives and inductively from concepts that emerged during early review of transcripts. This provided our analysis the benefits of previous research findings while creating space for us to “learn from [our] respondents, allowing for surprising results and phenomena to emerge” [22] (p. 7). NAB and EJG applied these index codes to the 52 de-identified interviews using Dedoose, then separately pulled all excerpts for each pertinent index code, and finally, applied a set of analytic codes developed in the same deductive-inductive approach as the index codes. By limiting text to only relevant excerpts, a more focused line-by-line analysis was possible, increasing the depth and breadth of our analysis [22]. We discussed and refined analytic code definitions multiple times during coding to ensure reliability and validity across interviews and additional codes were added when necessary. A sample of interviews were double coded and discrepancies resolved through discussion. Once analytic codes were applied, excerpts were pulled and analyzed for emerging themes. Particular attention was paid to negative cases that did not fit our explanations and conceptual framework.

## Results

Participants held degrees in nursing, medicine, pharmacy, and public health (Table 2). Administrators and prescribers had range of specialties, including internal medicine, family medicine, and surgery.

**Table 2:** Respondent Characteristics

***Characteristics***	***Administrators***	***Prescribers***
**Respondents** (#)	14	38
**Education**		
Physician	9	21
Physician Assistant	0	15
Nurse^a^	4	2
Pharmacist	1	0
Other^b^	5	0
**Specialty**^c^		
*Internal Medicine*	4	11
*Family Medicine*	4	9
*General Surgery*	0	3
*OB/GYN*	0	2
*Emergency Medicine*	0	7
*Psychiatry*	1	1
*Orthopedics*	0	3
*Otolaryngology (ENT)*	0	1
*Neurology*	0	1
*Anesthesia*	1	0
**Region**		
*Central/South Central*	8	20
*Eastern*	3	5
*Western/Northwestern*	3	13

^a^Nurse includes BSN, MSN, and DNP

^b^Multiple degrees may be held including PhD, non-clinical public health degrees

^c^Specialties are included only for those administrators with physician training

In our analysis we identified three important themes related to the implementation of state-mandated opioid prescribing limits: organizational communications, prescriber education, and changes to electronic medical record (EMR) systems. The findings from administrator interviews provide context for the prescriber findings, with both interview groups presented together.

### Organizational communications

Administrators used traditional (e.g. email, in-person meetings) and non-traditional (e.g. EMRs) avenues of communication to raise awareness and reinforce prescribing limits in accordance with the law. Most prescribers preferred email, in-person meetings, or some combination of the two, while a few prescribers mentioned the EMR system as a form of communication.

Prescribers who favored email mentioned the benefit of having something in writing to reference if needed; prescribers often favored succinct and easy to skim emails – a format that was less common. Prescribers expressed the drawbacks to email included the sheer number of such communications and concerns that important communications could get buried. An additional drawback was the inability to ask questions – an example question, lingering for full 2 years after passage of the law, asked by many prescribers during interviews was “How do opioid prescription refills fit in to the law?”

Prescribers, emphasizing the disadvantages of email, reflected on the benefits of in-person communication with the ability to raise important issues to the “surface.” Administrators agreed, noting that in-person communications allowed prescribers to voice their questions and concerns. Administrators also mentioned in-person gatherings could help increase prescriber “buy-in,” as described by an administrator when asked which communication method was most effective for communicating prescribing limits:

*A_118: I think the meetings for [when] all the providers are together and they can ask questions, and other people can support their experience. Whether they're academia background or community-based... If providers listen to other providers much more than they're going to listen to a clinical informatics person, ‘the EHR is changing, your order sets are changing, you're going to have an upgrade, you won't see this anymore, you're going to see this,’ they [get upset]. You need to sit down one on one, or have, where they have a group meeting and somebody has already done it and showed them how and the why. And then the provider will buy into it.*

This shows that in addition to providing space for asking questions, face-to-face communication allows providers to hear from their peers about changing hospital policies.

While in-person meetings had benefits, they were also labor intensive and demanded more of prescribers' limited time.

*A_124: I liked teaching face-to-face, that was good, but it was really hard to get face-to-face time because everybody's really busy … Again, we would do face-to-face, we'd have email blasts that would go to all the different departments. We would show up if somebody had a concern, we'd do hallway material and patient handouts, physician handouts, and yada, yada. Tried to kind of cover the bases.*

This administrator, who described themselves as an appointed opioid prescribing limits policy champion within their large healthcare system, notes that limited time makes it necessary to deliver information in varied ways. The importance of using multiple forms of communication to manage the benefits and drawbacks of each was echoed by other administrators. This helped to ensure prescribers were aware of prescribing limits even if they, for example, missed or disliked one form of communication.

Prescribers also supported using multiple channels of communication as an implementation strategy:

*P_216: There's constant emails and so you can get overwhelmed with changes, and all the different policies that go into effect at various times. So for me personally, probably having repeated information about what has to happen is helpful. So learning in different ways and using different tools to get the information across is helpful for me, so seeing it and then doing it and hearing it, for me*

This prescriber recognizes “repeated information” in varied forms is important, especially in light of the myriad changes to which prescribers are often subject. Given that the prescribers expressed various preferences for communications, noting benefits and drawbacks to each, and had concerns about keeping track of the many policy changes, the multiple channel communication approach by administrators is warranted with these types of policy changes.

### Prescriber and patient education

In their efforts to raise awareness and reinforce prescribing limits, administrators emphasized prescriber education and directed resources towards that. Although there were prescribers who reported not receiving any internal support, most prescribers reported receiving some type of internal support, typically in the form of education. The healthcare organizations generally provided educational resources through written materials (e.g. emails) and some presentations. These materials often emphasized prescribing limits from a legal standpoint—what the law is, when it was going into effect, and repercussions for not following it. As two prescribers noted in reference to prescribing limits’ communications:

*P_214: It was a required meeting for all the physicians. They [my organization] had multiple of them and they had someone coming to speak from the [NC] Medical Board. It was really implemented at that time. … [it] was more about the STOP Act. Prescribing limits. Penalties. It was definitely the stick. No carrot. I think the one from the [NC Medical Board] was required.*

*P_228: People were redundantly reminded and repeated and it was beaten in their head. You need to look at this, you need to review this, you need to read this, you need to see this, you need to sign off on this.*

Thus, following the law and what it would mean to not follow it was an important concern of healthcare administration according to prescribers; administrators provided this information in such a way that prescribers sometimes viewed it as a “stick” or “beaten” into their heads.

In contrast, clinical department support, meaning the departments inside of the healthcare system within which prescribers worked, took the form of presentations and meetings along with emails and often emphasized the clinical issues surrounding opioid prescribing specific to their medical specialties. These issues included alternative pain management options, addiction statistics, patient safety, and minimizing harm. Further, prescribers found presentations by pain management and pharmacy colleagues particularly helpful to learn about the finer details of prescribing limits, in-house opioid prescribing policies, and other pain management options. As one prescriber noted:

*P_237: If we didn’t have the resources of pain management position in there, then yes, it would [be difficult to comply with the prescribing limits] because, what are they [the patients] going to do?”*

Prescribers noted the need for effective ways to manage their patients’ pain whereas administrators were more focused on raising awareness and communicating about the law, in part because of a focus on compliance.

*A_119: … We do have the ability to monitor prescribing practices from our providers here in the hospital, and make sure that we are, if not 100% within those limits, mostly within those prescribing limits. And for folks who exceed the prescribing limits, we have an opportunity for one-on-one feedback.*

Prescribers repeatedly mentioned this need for support for alternatives to pain management, along with several mentions of the need for substance use treatment options – seemingly beyond the scope of an initial opiate prescription and notably absent from the NC STOP Act.

For some prescribers, internal support was lacking. Those who desired additional internal support wanted their organization to cast a wider educational net to address questions they had regarding prescribing limits (e.g., what happens if a patient needs a refill) and opioid prescribing in general. Prescribers also expressed a desire for clear organizational expectations concerning opioid prescribing and prescribing limits as well as organizational follow-up to discuss the progress of prescribing limits implementation. Prescribers wanted practical help such as “reminders” and people to take on the extra labor required. Most importantly, they desired education and trainings on communication strategies for discussing prescribing limits with patients:

*P_230: I would have liked them [administrators] to provide perhaps more support to clinicians as they're trying to explain the legislation and the policy so that you don't have to be, you know, it's like don't shoot the messenger. It was just basically you're kind of the punching bag if someone gets mad that you're not managing their pain the way they'd like you to manage it...I had questions … How to handle it if the patients gets upset with these limits that have been imposed on us.*

About one-third of prescribers said that training on how to communicate prescribing limits to patients would be useful for themselves and/or others; a belief echoed in administrator interviews.

*A_112: We didn't really do well with the patient education piece. That could have been better … Even just general opioid safety. I don't like the way we did it.*

*A_120: I think that speaking from a very simplistic standpoint, I'm sure that there's a lot of physicians that could use up to date education and up to date guidelines to help their practice. That's one thing that I see lacking in our areas. I'd like to see more information going to physicians to help them better make choices for their patients …*

Notably, when administrators were asked what advice they would give to other hospitals regarding prescribing limits implementation, the most common response was to focus on communication with and education of patients. Interestingly, a few prescribers also mentioned a need for improved patient education though who was responsible for delivering that education directly to patients was not abundantly clear.

Even though communication support may have been lacking, prescribers did describe receiving administrative support for other types of engagement with patients. Specifically, administrative support was reported with respect to patient complaints. A few prescribers explicitly referenced the era of the “pain as the fifth vital sign” that occurred during the late 1990s and early 2000s [23] when prescribers felt they were “reprimanded” by administrators when patients complained about inadequate pain control, now prescribers reported that administrators supported “appropriate clinical care” and would not throw them “under the bus” to manage patient complaints.

External support was typically through newsletters as well as formal continuing medical education (CME) courses and lectures at annual meetings and professional conferences. Prescribers differed on whether they received external support to implement opioid prescribing limits. For those that felt they were supported, prescribers noted the important role of professional organizations to learn about prescribing limits and legislative timelines, provide quick reference sheets [24], and discuss opioid prescribing on a more general level.

Administrators echoed these views, noting the role of outside government-supported organizations like NC Area Health Education Committee (AHEC) and specialty organizations like the North Carolina Healthcare Association (NCHA) and NC Medical Society in providing education.

*A_111 : We've done a ton of education. The medical school put together with, I think it was [large North Carolina city] AHEC , an intensive educational offering when the STOP Act was passed*

NC AHEC is an influential presence in the state with the core missions of provider education, practice change, and promoting clinical educational partnerships [25]. In addition, prescribers and administrators often mentioned the NC Medical Board (NCMB)’s STOP Act prescribing limits summary sheets and website as useful sources of information. The NCMB information stood out to prescribers; many of their EMR systems had links embedded where they could click to read the STOP Act details directly from the NCMB website.

External sources also played an important role in making prescribers aware of opioid prescribing limits legislation. While many prescribers first learned of prescribing limits within their practice organization, a roughly equal number did so externally through their professional organizations (e.g. NC Academy of Family Physicians), governing bodies, or CME activities. One administrator from a larger institution emphasized the importance of external sources being other hospitals with whom they shared information regarding strategies for implementing opioid prescribing limits in the clinical setting:

*A_113: we have talked to other people from other medical centers around the state who don't have those resources in play, and they've been asking, “Could you just give us the recommendations that you have at least?” Then try to implement those, like [Hospital X], and [Hospital Y] in the western part of the state.*

Indeed a few administrators discussed leveraging their networks and partnerships with peer institutions for this purpose – a way for larger institutions to share resources (e.g. formal trainings) with smaller ones.

### Changes to the EMR systems

Nearly all administrators and prescribers mentioned changes to the EMR in response to the enactment of the prescribing limits. Administrators leveraged a “remind clinicians” strategy [13] using EMR prompts and alerts to implement prescribing limits. These EMR alerts are commonly classified as (1) passive alerts, where prescribers can move forward without responding, (2) soft-stops, which allow for prescribers to close them without making any decisions, (3) hard-stops, where a prescriber must respond in order to move forward, and (4) pre-filled defaults, where typically a prefilled amount was the equivalent of 3, 5, or 7 days of opioids [26].

Prescribers described EMR alerts (also referred to as “pop-ups” in interviews) as informational (e.g. links to prescribing limits legislation), acknowledgements (e.g. are you in compliance with STOP Act? Check yes/no), or warnings (e.g. you are above the prescribing limit). For those who used EMR alerts, administrators described them as important tools for both raising awareness and reminding prescribers of the STOP Act prescribing limits:

*Interviewer: When you think about the STOP Act and those outliers, do you think that there are physicians who may not be aware of the STOP Act?*

*A_116: Yes. So we put the alerts in our electronic medical records so they cannot prescribe for more than that. What happens is if you have electronic medical record systems, the prescription of written in that one, it puts a stop on how much you can prescribe, how many days of medication you can prescribe from the [Emergency Department]. So it helps them and then it gives you an alert if you want to do it more and tells you about the STOP Act.*

*Interviewer: Where did that come from? That alert?*

*A_116: That alert was generated as the STOP Act was coming. Because still you cannot get every physician. But if you put some sort of guardrail around where they're prescribing, at the time of prescribing, it helps.*

This administrator demonstrates the lack of clarity on how EMR alerts operated. Indeed, this administrator mentions “alerts” that do not allow for more to be prescribed, which indicates more than a simple message but rather a hard limit. Administrators often conflated terms and switched among them in their responses. They typically described an alert or soft-stop that would help prescribers, but in practice many of the EMR changes for implementing prescribing limits appeared to be hard-stops, regardless of the terminology used by either group. For example:

*P_217: I know we get a lot of emails pertaining to the STOP Act. Whenever we are prescribing opioids through our charting system, there is a flag. I don't know if that was actually instated by the STOP Act or if that was through [Hospital System]. But it flags it and says, “Hey, per the STOP Act you're only allowed to do this much. Are you compliant with this?” We have to click yes or no, in order to be able to prescribe it.*

This prescriber describes the alert as a “flag” where “we have to click yes” indicating the hard-stop. Prescribers noted that passive alerts and hard-stops could be helpful by: reminding them what the prescribing limits were (particularly for those who prescribe infrequently), providing important links to the legislation itself, and creating space for prescribers to pause and “think” before completing a prescription. Furthermore, hard-stops obligated them to be compliant:

*P_231: Having that there as a little tickler or reminder, and that it requires you to be compliant with the law because you can't order anything that is outside of the law limits, outside of the limits of the law. So that's our whole system, it's not just my family practice but the whole system is built that way. So yeah, I would say that's easier.*

They note that the hard-stop is a reminder of the prescribing limits law that makes it “easier,” meaning less of burden to remember what the limits are. In contrast, other prescribers found EMR changes for implementing prescribing limits lacking. This was due in part to the abundance of alerts in the EMR which caused some alerts to “get lost” among the “noise”:

*P_215: I think with the EMR we're so trained to click through things that often those types of messaging that's very important can get lost in the background noise. Whereas if I have an email that's more explicit to remind me of these things I'm in a different mindset when I'm reading that versus looking through the EMR to accomplish a task for a patient or a patient encounter. I think I definitely get more out of reading an email.*

This prescriber’s view of EMR alerts as having limited effect on their clinical decision-making suggests no singular response to the IT changes. Indeed, prescribers demonstrated no global preference for using (or not using) EMR alerts to implement prescribing limits.

A second EMR-based strategy for implementation was setting pre-filled prescription defaults. In some instances, the pre-filled default number of days can be changed as this prescriber describes:

*P_203: … when the prescription is being written, I write my prescriptions electronically and send them electronically through that double verification system. And generally, when I write them and put the dose or the type of narcotic, it will auto-populate. A lot of times I'll go back and just fill it in the way I want it, so I'll give a certain number of days of prescription to the patient depending on what they've had done.*

Furthermore, the pre-filled default is an indication of healthcare organizations’ “expectations” and a communication of policy:

*Interviewer: In your understanding, how does the hospital's policy differ from the prescribing limits in the NC STOP act?*

*P_236: I don't think they do. I think we are following it. I think the expectation is that all of us who prescribe are following that legislation. … I don't know that anyone's watching this individually, but the expectation is that we are following the law and at any time the NC controlled substance. People can check to see if we are and we can be held accountable if we are not*

*Interviewer: What makes you say that about expectations? I guess I'm wondering*

*P_236: Because, when you go to write a prescription for a patient, it defaults to what the rules are. And I want to think that we actually have had to sign, not an agreement, but some sort of form that says we recognize best practices related to opioid prescribing this limitation of the legislation.*

This exemplifies comments from others that the default in the EMR system (along with a signed agreement) was a “rule” set by hospital leadership, a stance on prescribing limits assumed to be in line with what was codified in the STOP Act. It is notable that this same prescriber learned, in the course of the interview, that the STOP Act prescribing limit for acute pain patients was different (5 days), from the default setting (3 days) in their EMR system:

*P_236: It's interesting that I, for a while had thought [prescribing limits] was only three days on the acute pain and that being what is sort of the default on my institution's prescribing page that comes up.*

Thus, changes in the EMR system were not only seen as reminders, but viewed by some prescribers as evidence of an organizational policy even when it may be more restrictive than the legislation. This echoed administrators’ focus on the EMR as a tool for implementation, but reflected different understandings of the meaning behind those changes. For example, this administrator mentions EMR alerts as “information” for the prescribers:

*A_117: Something that is also going live this week is whenever they order opioids, inpatient or whenever, it's going to throw a little box up that tells them what are the morphine equivalents that they're prescribing. They're going to start to get warnings, I guess it's more information purposes, but it's not going to block them. It's just more of a, "This is what the best practice is.”*

Administrators largely expressed positivity for their approaches to implementation of prescribing limits using the EMR system, rarely viewing such changes as demonstrating the healthcare organization’s own policy and expectations. Instead these changes were a way to support prescribers in their own clinical decision-making.

## Discussion

Hospital administrators sought to raise awareness and reinforce knowledge of the STOP Act prescribing limits through different implementation strategies including reminding physicians of the mandate using EMR system changes and distributing educational materials for prescribers. Prescribers did not have a unified preference for these different forms of communication, which reinforced administrators’ multi-channel approach. This approach, however, called attention to a dilemma: by using so many communication channels, administrators burden the prescriber with tracking different sources of information and interpreting the embedded expectations when clinical time is precious. Indeed, because many prescribers do not practice healthcare in terms of what is legal or illegal, some modes of information (e.g. email) may make it more difficult to discern between policy mandates (a requirement) versus best practice guidelines (e.g., the CDC opioid prescribing guidelines) versus organizational preferences [27]. Communication strategies that improve prescriber knowledge of when a state mandate is applicable and support in identifying these distinctions are pivotal for ensuring compliance [28].

Administrators leveraged EMR-based strategies to “guide” prescribers rather than “enforce” the law, using terms such as “alerts” and “reminders” even when describing hard-stops. However, the administrators were implementing a mandate, not a guideline, with a definitive date for legal compliance. Given the legality, these strategies signaled an organizational policy and/or the legislation itself, putting administrators in the unexpected role of sharing responsibility for clinical decision-making. If prescribers had questions about the implementation of prescribing limits, the ability to have those questions answered were constrained by the realities that, despite limited evidence of enforcement of prescribing limits, prescribers are fearful of losing their licenses if their prescriptions are perceived as inadequate or excessive. Our findings showed that many prescribers viewed EMR changes as beneficial, in part because when they had difficulty recalling the specifics of the limits, the EMR “rules” would protect them from breaking the law. A drawback of the EMR-based strategy, however, was the inability to make adjustments, tailor to a patient’s needs or simply ask for clarifications in the moment; such rigidity within the EMR may reflect the rigidity of the mandate or, perhaps more accurately, the perceived rigidity as interpreted by healthcare systems. Further exploration of reactions, including better understanding of the specialty differences and different patient populations being served, may give insight in to how such EMR strategies have differential impacts.

For prescribers’ education, administrators and prescribers described an array of resources and reinforced the importance of face-to-face interactions. Prescriber education provided by administrators and the healthcare systems tended to focus on the legal aspects of prescribing limits, emphasizing the importance of compliance while prescribers’ own departments were more tailored to address the clinical aspects of prescribing limits by, for example, discussing pain management alternatives (Additional file 2). External resources and partners including professional societies, NC AHEC, and the NCMB also played a key role in education by providing information and prescriber trainings. Our findings on external support echo the research of others – Stone and colleagues found that strategies for implementing prescribing limits mandates largely focus on awareness through materials generated by government agencies (e.g. the state health department) and targeted education conducted by state professional societies [29].

Although opioid prescribing limits legislation centers on prescriber behavior, administrators and prescribers desired more resources for both interactions with and education of the patient community. This begs the question, “Who should provide this education?” While providers are educated by their own healthcare systems, their professional societies, the NCMB, and NC AHEC, there is a need to determine who is responsible for educating the general population about opioid policy and ensuring it is in line with prescriber expectations and practices. In making such determinations of responsibility and, ultimately, preparing patients to be active participants in prescribing limits implementation, may go a long way in improving prescriber uptake.

Future research should assess the efficacy and acceptability of communication strategies used to implement mandated opioid policies. Measures of acceptability, a key implementation outcome defined as the “perception among implementation stakeholders that a given treatment, service, practice, or innovation is agreeable, palatable, or satisfactory” [30], have been widely tested in the behavioral health and substance use health services literature [31]. However, a legal mandate may not allow for tailoring to meet the needs of different specialties, thus requiring different considerations when it comes to provider acceptability. Furthermore, tailoring to ensure prescribers serving patients who are not acute pain patients are not inadvertently impacted is critical; recent research suggests that government-driven opioid prescribing limits have negatively impacted persons with chronic pain and cancer pain [29, 32–34]. This also highlights the need for better understanding of how healthcare-based implementation of opioid prescribing limits legislation may further exacerbate health inequities and the need to measure success of policy implementation in a more patient-centered way [35].

This study is subject to a few limitations. The societal conversation of the “opioid epidemic” in the United States, strategies to curb opioid overdoses, and other provisions in the STOP Act were, at times, difficult to disentangle from participant perspectives on implementation of prescribing limits legislation. Many of the interviewees referred to their experiences with chronic pain patients, the “opioid epidemic,” and persons in need of substance use treatment when responding to questions. They also mentioned other provisions of the STOP Act that focused on chronic pain patients and strengthening prescription drug monitoring programs (PDMPs). We made every effort to remind interviewees our focus was on prescribing limits as codified in the STOP Act and excluded excerpts from the data analysis where discussions became too muddled with other STOP Act provisions or broader societal issues, yet entanglement with these influences cannot be fully excluded and reflect the importance of considering the external factors outside of the healthcare system control when discussing policy implementation [36]. Indeed, over and over again prescribers did not view opioid prescribing limits as separate from PDMPs or treatment but rather all intertwined - a finding in and of itself from our analysis. In addition, at the end of our data collection period the United States healthcare workforce began responding to the global pandemic of COVID-19 (March 2020). Given our participant population of healthcare workers, we ceased data collection and pivoted to analysis. Ultimately, we believe that the data we collected on experiences and reflections on different strategies of implementation were well rounded in our interviews and thus do not feel that our conclusions are lacking from incomplete data.

## Conclusions

Administrators and prescribers across NC used a range of strategies to implement the 2017 NC STOP Act prescribing limits. A statewide legal mandate with limited guidance on implementation resulted in piecemeal approaches and a range of experiences. Hospital administrators were primarily focused on strategies that ensured their organizations followed the law, with limited focus on how such strategies were received by prescribers or what concerns they may have with impacts on patient care. Prescribers were focused on strategies that adequately manage patient pain -- providing good healthcare – while working to interpret governmental and healthcare system expectations when a mindset of “what is legal” was more foreign to them.

As states continue to wrestle with how to legislate responses to widespread opioid overdose and death, understanding the NC experience in implementing prescribing limits informs how states might improve upon how laws are written and how healthcare systems might communicate with as well as support prescribers and patients more effectively. Future research should explore prescriber acceptability and effectiveness of existing implementation strategies, particularly as integrated into EMR systems, and identify new implementation strategies as state laws on opioids continue to evolve.

## Supplementary Information

**Additional file 1.** SRQR Guidelines for Reporting Qualitative Research Studies.

**Additional file 2.** Key Themes of Organizational Communications, Prescriber and Patient Education, and Changes to the EMR System.

## Data Availability

The datasets used and/or analyzed during the current study are available from the corresponding author on reasonable request.

## Abbreviations

CFIR: Consolidated Framework for Implementation Research
MD: Medical doctor
NP: Nurse Practitioner
PA: Physician’s Assistant
STOP Act: Strengthen Opioid Misuse Prevention Act

## References

[1] Bohnert AS, Valenstein M, Bair MJ, Ganoczy D, McCarthy JF, Ilgen MA, Blow FC (2011). Association between opioid prescribing patterns and opioid overdose-related deaths. JAMA..

[2] Makary MA, Overton HN, Wang P. Overprescribing is major contributor to opioid crisis. BMJ. 2017;359:j4792. 10.1136/bmj.j4792. 10.1136/bmj.j4792 29051174

[3] Schirle L, Stone AL, Morris MC, Osmundson SS, Walker PD, Dietrich MS, Bruehl S (2020). Leftover opioids following adult surgical procedures: a systematic review and meta-analysis. Syst Rev.

[4] Chua K-P, Kimmel L, Brummett CM (2020). Disappointing early results from opioid prescribing limits for acute pain. JAMA surgery.

[5] Dowell D, Haegerich TM, Chou R (2016). CDC guideline for prescribing opioids for chronic pain—United States, 2016. JAMA..

[6] McGinty EE, Stuart EA, Alexander GC, Barry CL, Bicket MC, Rutkow L (2018). Protocol: mixed-methods study to evaluate implementation, enforcement, and outcomes of US state laws intended to curb high-risk opioid prescribing. Implement Sci.

[7] Fink BC, Uyttebrouck O, Larson RS (2020). An effective intervention: limiting opioid prescribing as a means of reducing opioid analgesic misuse, and overdose deaths. J Law Med Ethics.

[8] Davis CS, Piper BJ, Gertner AK, Rotter JS (2020). Opioid prescribing laws are not associated with short-term declines in prescription opioid distribution. Pain Med.

[9] Abuse NIoD (2019). Opioid summaries by state: NIH.

[10] Ahmad F, Rossen L, Sutton P (2020). Provisional drug overdose death counts.

[11] House NC (2017). HB 243, Strengthen Opioid Misuse Prevention (STOP) Act 2017 [updated 6/29/2017].

[12] Proctor EK, Powell BJ, McMillen JC (2013). Implementation strategies: recommendations for specifying and reporting. Implement Sci.

[13] Powell BJ, Waltz TJ, Chinman MJ, Damschroder LJ, Smith JL, Matthieu MM, Proctor EK, Kirchner JAE (2015). A refined compilation of implementation strategies: results from the expert recommendations for implementing change (ERIC) project. Implement Sci.

[14] Heidbreder EG (2017). Strategies in multilevel policy implementation: moving beyond the limited focus on compliance. J Eur Public Policy.

[15] Hamilton AB, Finley EP (2019). Qualitative methods in implementation research: an introduction. Psychiatry Res.

[16] Liu JB, Kelz RR (2018). Types of Hospitals in the United States. JAMA.

[17] Palinkas LA, Horwitz SM, Green CA, Wisdom JP, Duan N, Hoagwood K (2015). Purposeful sampling for qualitative data collection and analysis in mixed method implementation research. Adm Policy Ment Health Ment Health Serv Res.

[18] Chayama KL, McNeil R, Shoveller J, Small W, Knight R (2020). Implementation opportunities and challenges identified by key stakeholders in scaling up HIV treatment as prevention in British Columbia, Canada: a qualitative study. Implement Sci Commun.

[19] Ringwalt C, Gugelmann H, Garrettson M, Dasgupta N, Chung AE, Proescholdbell SK, Skinner AC (2014). Differential prescribing of opioid analgesics according to physician specialty for Medicaid patients with chronic noncancer pain diagnoses. Pain Res Manag.

[20] Damschroder LJ, Aron DC, Keith RE, Kirsh SR, Alexander JA, Lowery JC (2009). Fostering implementation of health services research findings into practice: a consolidated framework for advancing implementation science. Implement Sci.

[21] O’Brien BC, Harris IB, Beckman TJ, Reed DA, Cook DA (2014). Standards for reporting qualitative research: a synthesis of recommendations. Acad Med.

[22] Deterding NM, Waters MC. Flexible coding of in-depth interviews: a twenty-first-century approach. Sociol Methods Res. 2018. 10.1177/0049124118799377.

[23] Jones MR, Viswanath O, Peck J, Kaye AD, Gill JS, Simopoulos TT (2018). A brief history of the opioid epidemic and strategies for pain medicine. Pain Ther.

[24] North Carolina Medical Board N. STOP Act Overview Raleigh, NC 2017 [Available from: https://www.ncmedboard.org/images/uploads/article_images/STOPACT-onepager.pdf. Accessed 11 Nov 2020.

[25] About us: NC AHEC Chapel Hill, NC: NC AHEC; 2020 [Available from: https://www.ncahec.net/about-nc-ahec/about-us/. Accessed 6 Feb 2021.

[26] Powers EM, Shiffman RN, Melnick ER, Hickner A, Sharifi M (2018). Efficacy and unintended consequences of hard-stop alerts in electronic health record systems: a systematic review. J Am Med Inform Assoc.

[27] Dennis S, Waterworth S (2021). Health Professionals’ engagement with email—enabler or disrupter?. Comput Inform Nurs.

[28] Keyworth C, Hart J, Armitage C, Tully M (2018). What maximizes the effectiveness and implementation of technology-based interventions to support healthcare professional practice? A systematic literature review. BMC Med Inform Dec Mak.

[29] Stone EM, Rutkow L, Bicket MC, Barry CL, Alexander GC, McGinty EE (2020). Implementation and enforcement of state opioid prescribing Laws. Drug Alcohol Depend.

[30] Proctor E, Silmere H, Raghavan R, Hovmand P, Aarons G, Bunger A, Griffey R, Hensley M (2011). Outcomes for implementation research: conceptual distinctions, measurement challenges, and research agenda. Adm Policy Ment Health Ment Health Serv Res.

[31] Mettert K, Lewis C, Dorsey C, Halko H, Weiner B (2020). Measuring implementation outcomes: an updated systematic review of measures’ psychometric properties. Implement Res Pract.

[32] Lajam CM, Cenname J, Hutzler LH, Bosco JA (2019). Ethics of opioid prescriber regulations: physicians, patients, and pain. JBJS..

[33] Rubin R (2019). Limits on opioid prescribing leave patients with chronic pain vulnerable. JAMA..

[34] Ranapurwala SI, Ringwalt CL, Pence BW, Schiro S, Fulcher N, McCort A, ... Marshall SW. State medical board policy and opioid prescribing: a controlled interrupted time series. Am J Prev Med. 2021;60(3):343–51. 10.1016/j.amepre.2020.09.015. 10.1016/j.amepre.2020.09.015 PMC7902466 33309449

[35] Kertesz SG, McCullough MB, Darnall BD, Varley AL (2020). Promoting patient-centeredness in opioid Deprescribing: a blueprint for De-implementation science. J Gen Intern Med.

[36] Watson DP, Adams EL, Shue S, Coates H, McGuire A, Chesher J, Jackson J, Omenka OI (2018). Defining the external implementation context: an integrative systematic literature review. BMC Health Serv Res.

