# Supplementary material for: Implementation of mandatory opioid prescribing limits in North Carolina: healthcare administrator and prescriber perspectives
Source: BMC Health Serv Res. 2021 Nov 3;21:1191. doi: 10.1186/s12913-021-07230-5 (PMC8565171; doi:10.1186/s12913-021-07230-5)
Supplement: Supplementary file 1 — Additional file 1. SRQR Guidelines for Reporting Qualitative Research Studies. [file 12913_2021_7230_MOESM1_ESM.pdf]

Additional File 1. SRQR Guidelines for Reporting Qualitative Research Studies

O'Brien BC, Harris IB, Beckman TJ, Reed DA, Cook DA. Standards for reporting qualitative research: a synthesis of recommendations. Acad Med. 2014;89(9):1245-1251.

	Reporting Item	Page Number
**Title**		
	Concise description of the nature and topic of the study identifying the study as qualitative or indicating the approach (e.g. ethnography, grounded theory) or data collection methods (e.g. interview, focus group) is recommended	2
**Abstract**		
	Summary of the key elements of the study using the abstract format of the intended publication; typically includes background, purpose, methods, results and conclusions	2
**Introduction**		
Problem formulation	Description and significance of the problem / phenomenon studied: review of relevant theory and empirical work; problem statement	3
Purpose or research question	Purpose of the study and specific objectives or questions	3
**Methods**		
Qualitative approach and research paradigm	**Qualitative approach** (e.g. ethnography, grounded theory, case study, phenomenology, narrative research) and guiding theory if appropriate; **identifying the research paradigm** (e.g. postpositivist, constructivist / interpretivist) is also recommended; **rationale**. The rationale should briefly discuss the justification for choosing that theory, approach, method or technique rather than other options available; the assumptions and limitations implicit in those choices and how those choices influence study conclusions and transferability. As appropriate the rationale for several items might be discussed together.	4-5
Researcher characteristics and reflexivity	Researchers' characteristics that may influence the research, including personal attributes, qualifications / experience, relationship with participants, assumptions and / or presuppositions; potential or actual interaction between researchers' characteristics and the research questions, approach, methods, results and / or transferability	6, 20
Context	Setting / site and salient contextual factors; rationale	4-6

Sampling strategy	How and why research participants, documents, or events were selected; criteria for deciding when no further sampling was necessary (e.g. sampling saturation); rationale	4
Ethical issues pertaining to human subjects	Documentation of approval by an appropriate ethics review board and participant consent, or explanation for lack thereof; other confidentiality and data security issues	6
Data collection methods	Types of data collected; details of data collection procedures including (as appropriate) **start and stop dates of data collection and analysis**, iterative process, triangulation of sources / methods, and modification of procedures in response to evolving study findings; rationale	6
Data collection instruments and technologies	Description of instruments (e.g. **interview guides, questionnaires**) and **devices** (e.g. audio recorders) used for data collection; if / how the instruments(s) changed over the course of the study	4-6
Units of study	Number and relevant characteristics of participants, documents, or events included in the study; level of participation (could be reported in results)	4, 7
Data processing	Methods for processing data prior to and during analysis, including transcription, data entry, data management and security, verification of data integrity, data coding, and anonymisation / deidentification of excerpts	6-7
Data analysis	Process by which inferences, themes, etc. were identified and developed, including the researchers involved in data analysis; usually references a specific paradigm or approach; rationale	6-7
Techniques to enhance trustworthiness	Techniques to enhance trustworthiness and credibility of data analysis (e.g. member checking, audit trail, triangulation); rationale	6-7
**Results/findings**		
Syntheses and interpretation	Main findings (e.g. interpretations, inferences, and themes); might include development of a theory or model, or integration with prior research or theory	7-16
Links to empirical data	Evidence (e.g. quotes, field notes, text excerpts, photographs) to substantiate analytic findings	7-16
**Discussion**		
Integration with prior work, implications, transferability and contribution(s) to the field	Short summary of main findings; explanation of how findings and conclusions connect to, support, elaborate on, or challenge conclusions of earlier scholarship; discussion of scope of application / generalizability; identification of unique contributions(s) to scholarship in a discipline or field	16-18

Limitations	Trustworthiness and limitations of findings	17-18
**Other**		
Conflicts of interest	Potential sources of influence of perceived influence on study conduct and conclusions; how these were managed	19
Funding	Sources of funding and other support; role of funders in data collection, interpretation and reporting	19
