# Supplementary material for: Implementation of mandatory opioid prescribing limits in North Carolina: healthcare administrator and prescriber perspectives
Source: BMC Health Serv Res. 2021 Nov 3;21:1191. doi: 10.1186/s12913-021-07230-5 (PMC8565171; doi:10.1186/s12913-021-07230-5)
Supplement: Supplementary file 2 — Additional file 2. Key Themes of Organizational Communications, Prescriber and Patient Education, and Changes to the EMR System. [file 12913_2021_7230_MOESM2_ESM.pdf]

Additional File 2

**Additional File 2**. Key Themes of Organizational Communications, Prescriber and Patient Education, and Changes to the EMR System

| Theme | Administrators | Prescribers |
|---|---|---|
| **Organizational Communications** | | |
| | A_112: Now did people read [our organizational email about the STOP Act]? I don't know. I've done tons and tons of training outreach. We have online training. I've been to many, many divisions, departments, given one hour presentations… their faculty meetings, whatever. Others have done this too but I've been the kind of main person doing that. So there's a lot of going out and talking to orthopedics, primary care, radiation oncology, whatever.<br><br>A_124: Case-by-case, department-by-department. We would just go out and talk to [the prescribers], and trying to find ways that everybody could win.<br><br>A_120: I don't think that [the mandated prescribing limits] have a significant impact on operations. I think that we've asked physicians and providers to do business differently and it's been some time from that. At this point I haven't heard any complaints.<br><br>A_114: We also spell out the specific requirements, for example, with the surgeons. You can only provide X amount of opioids after a surgical procedure… Our surgeons are aware of that ahead of time and everything.<br><br>A_121: When [the STOP Act] first came out, I'll be honest, it was a little bit confusing. It felt like there wasn't, I think, quite enough information about how to enact it appropriately. Really kind of making sure that ... I don't know if there was enough time spent in really making sure we all understood when to apply each set of guidelines. It was just an email that said, "Hey, there's a new law, five days or seven days," and that was kind of the extent of the education provided | P_212: There's not a hard stop, but it's one of those expected things. It's one of the things that our health system has made a metric that they're watching, and that we're being evaluated on. I think it's something that's not necessarily the group, but the bigger picture that has set guidelines for us as well.<br><br>P_217: I know we get a lot of emails pertaining to the STOP Act. Whenever we are prescribing opioids through our charting system, there is a flag.<br><br>P_223: My attending just says, "I trust you to make the right decisions," so it's kind of just an oral thing, communications. I don't usually pass it by him. I just do what I feel is best for the patient.<br><br>P_202: Patient satisfaction was a driving impetus for administrative involvement in the clinical practice of medicine, and if they felt that the complaint was based upon not receiving the patient's requested number of narcotics, they would reprimand individuals for not providing that. Now that practice has changed, and I've not heard anybody receiving reprimands for appropriate clinical care. Which includes limiting narcotic use.<br><br>P_225: We were told to tell [patients] the information about the STOP Act and also that if we got to the point that we were not able to talk to the patient and still being professional essentially, that they needed to speak with the patient relations, and then if necessary, get the administration involved. |
| **Prescriber and Patient Education** | | |
| | A_112: We have not done training of doctors on [communications of opioid prescribing limits to patients] and I have to say that it's not... I don't think people find that a difficult conversation. | P_211: I get a great deal of education from my pain management colleagues, because I don't know second options that I have for management of the pain such as in a non-medical case. So they do kind of educate me quite frequently about things that I could do and things that I could have done. |

| | | |
|---|---|---|
| | A_117: One of our hospitalists is, she's on this [County] Coalition for Opioid Abuse. She does a lot of provider and clinician education, has done a lot in the past two or three years to make providers aware of the problems. We have brought in people from Chapel Hill, as a matter of fact, to do programs on opioids and abuse.<br><br>A_117: We have brought in people from [Large NC Town], as a matter of fact, to do programs on opioids and abuse… Actually, it's usually done at, we don't really have space here large enough to have a big group, so it's usually done at one of the local restaurants or something like that that has a big meeting area.<br><br>A_118: If providers listen to other providers much more than they're going to listen to a clinical informatics person, the EHR is changing, your order sets are changing, you're going to have an upgrade, you won't see this anymore, you're going to see this, they [get upset]. You need to sit down one on one, or have, where they have a group meeting and somebody has already done it and showed them how and the why. And then the provider will buy into it. But it's always what's in it for me because a provider is incredibly busy anyway.<br><br>A_111: Physicians and clinicians are very motivated by peers' behaviors, so really having champions that act as examples for others to get onboard with, and follow, and then you have to keep the patient in mind, hence what I said about not halting prescribing, but making it in more precision in nature. | P_235: If we have patients that are complaining about extra pain and they want a refill early or they want something stronger and they're frustrated they have to call for refills rather than just get one script for more medicine at the same time, I will kind of explain to them that there's a state law and they kind of limit how much we're allowed to give them. And that seems to usually kind of cover it enough for them.<br><br>P_215: I think [additional training on patient education would be helpful] because there are times when it can be challenging when patients feel like... They can misinterpret a shorter duration of a prescription as either someone minimizing their pain, not hearing them, not understanding, or trying to withhold something of a mis-value, whether or not that's accurate, to them. I think being trained up front, feeling more comfortable having that conversation would be nice… a training would be really beneficial.<br><br>P_230: I would have liked them to provide perhaps more support to clinicians as they're trying to explain the legislation and the policy so that you don't have to be, you know, it's like don't shoot the messenger. It was just basically you're kind of the punching bag if [a patient] gets mad that you're not managing their pain the way they'd like you to manage it...I had questions…How to handle it if the patients gets upset with these limits that have been imposed on us. |
| **Changes to the EMR system** | | |
| | A_116: Yes [there are physicians who may not be aware of the STOP Act]. So we put the alerts in our electronic medical records so they cannot prescribe for more than that… What happens is if you have electronic medical record systems. The prescription of written in that one, it puts a stop on how much you can prescribe, how many days of medication you can prescribe from the ED. So it helps them and then it gives you an alert if you want to do it more and tells you about the STOP Act… That alert was generated as the STOP Act was coming. Because still you can not get every physician. But if you put some sort of guardrail around where they're prescribing, at the time of prescribing, it helps. | P_213: [The EMR alerts] get my attention, because like I say, I probably would've ignored the emails. They make me step back and think what I'm going to do, and to be honest it also at this point I've gotten so used to [the alerts] I'm already thinking about it but it also gives me an excuse to not give more. Because I can just say to the patient, "Listen, my hands are tied. This is as much as we can do right now. We'll have to chat with you after.<br><br>P_231: Having [the EMR alert] there as a little tickler or reminder, and that it requires you to be compliant with the law because you can't order anything that is outside of the law limits, outside of the |

| | | |
|---|---|---|
| | A_119: I think we can do more for sure on supporting providers and educating providers, keeping them up to date, those types of things. I think we could do more as an organization relative to that.<br><br>A_115: So, [e-prescribing] is a requirement as of January 2020 that we send controlled substances ... prescriptions electronically and I think that most pharmacists are accepting those now and most providers, in large, are set up for that. We're missing a component that we had to upgrade our EMR a couple months ago and now we're waiting for that component and there's a bunch of people in line for it. So we need to do that before we can switch [e-prescribing] on. | limits of the law. So that's our whole system, it's not just my family practice but the whole system is built that way.<br><br>P_236: The most I will prescribe is 12 tablets. There's actually a restriction on our EHR. And that's, that's the law. And I often, depending on the situation, I will even change that to a lower number sometimes<br><br>P_237: So I guess the biggest hassle for me is just having to sit down and manually write out, or enter all the data [in the EMR]. It takes me 21 clicks to write one opioid prescription. |
